# The chloroplast genome sequence and phylogenetic analysis of *Apocynum venetum* L.

**DOI:** 10.1371/journal.pone.0261710

**Published:** 2022-03-10

**Authors:** Xiaonong Guo, Zhuanxia Wang, Deyu Cai, Lei Song, Jialin Bai

**Affiliations:** 1 Key Laboratory of Biotechnology and Bioengineering of State Ethnic Affairs Commission, Biomedical Research Center, Northwest Minzu University, Lanzhou, China; 2 College of Life Science and Engineering, Northwest Minzu University, Lanzhou, China; 3 China-Malaysia National Joint Laboratory, Biomedical Research Center, Northwest Minzu University, Lanzhou, China; Huazhong University of Science and Technology, CHINA

## Abstract

*Apocynum venetum* L. (*Apocynaceae*) is valuable for its medicinal compounds and fiber content. Native *A*. *venetum* populations are threatened and require protection. Wild *A*. *venetum* resources are limited relative to market demand and a poor understanding of the composition of *A*. *venetum* at the molecular level. The chloroplast genome contains genetic markers for phylogenetic analysis, genetic diversity evaluation, and molecular identification. In this study, the entire genome of the *A*. *venetum* chloroplast was sequenced and analyzed. The *A*. *venetum* cp genome is 150,878 bp, with a pair of inverted repeat regions (IRA and IRB). Each inverted repeat region is 25,810 bp, which consist of large (LSC, 81,951 bp) and small (SSC, 17,307 bp) single copy areas. The genome-wide GC content was 38.35%, LSC made up 36.49%, SSC made up 32.41%, and IR made up 43.3%. The *A*. *venetum* chloroplast genome encodes 131 genes, including 86 protein-coding genes, eight ribosomal RNA genes, and 37 transfer RNA genes. This study identified the unique characteristics of the *A*. *venetum* chloroplast genome, which will help formulate effective conservation and management strategies as well as molecular identification approaches for this important medicinal plant.

## Introduction

*Apocynum venetum* L. (*Apocynaceae*) (Luobuma in Chinese) is a perennial herb distributed in Eurasia from Southeast Europe to Northern China. It occurs in floodplains and valleys along rivers such as the Tarim River [1, 2]. The roots, stems, leaves, and flowers of *A*. *venetum* have medicinal uses [3, 4] and these uses were documented in the “Compendium of Materia Medica.” In 1977, *A*. *venetum* was listed in the Pharmacopoeia of the People’s Republic of China as a primary treatment for hypertension and hyperlipidemia [5–8], and pharmacological studies have demonstrated that *A*. *venetum* possesses many pharmacological activities including cardiotonic [9], hepatoprotective [10, 11], antioxidant [12–14], antidepressant and anxiolytic effects [15–18]. *A*. *venetum* maybe useful for the prevention and treatment of cardiovascular and neurological diseases such as high blood pressure, high cholesterol, neurasthenia, depression, and anxiety [19–23].

*A*. *venetum* has relatively high salt tolerance, cold tolerance, drought tolerance, high temperature tolerance, and wind resistance [24, 25]. It is an important plant for the wind proofing and sand-stabilization of desert grasslands in Central Asia. *A*. *venetum* therefore combines ecological benefits and economic benefits [24, 26]. Overharvesting of wild *A*. *venetum* and environmental degradation have reduced *Apocynum* populations and protection of *Apocynum* germplasm resources is needed. Studies of *A*. *venetum* have mainly focused on its medicinal effects and physiological characteristics such as photosynthesis and water absorption [27, 28]. However, there are few studies on the genetic diversity and genetic structure of wild *A*. *venetum* populations [29, 30].

Chloroplasts (cps) are the descendants of ancient bacteria endosymbionts. They are important organelles in plant cells that are responsible for photosynthesis and other aspects of metabolism [31]. Cp DNA is independent of the nuclear genome and exhibits semi-autonomous genetic characteristics. The characteristics of maternal and highly conserved genes in the cp genome are favorable for studying plant phylogeny [32, 33]. Molecular barcodes based on the cp genome have potential for species identification, especially among closely related taxa [34, 35]. The complete cp genome sequence may provide reliable barcodes for accurate plant identification at species and population levels [36, 37]. In higher plants, photosynthesis occurs in cp, which provides the necessary energy for plant growth and survival.

There are many counterfeit *A*. *venetum* products on the market, and they are difficult to detect based on appearance. There is a need for a molecular method to distinguish counterfeit products. DNA barcode sequence analysis is a molecular identification technology that uses standardized DNA sequence fragments to provide a fast, accurate, and automated species identification method [38–41]. The non-coding region of the cp has been successfully used in research on the DNA barcode. *A*. *venetum* cp genome information can provide candidate DNA barcodes for the identification of *A*. *venetum* and counterfeit products.

In this study, we assembled and analyzed the *A*. *venetum* cp genome sequence based on Illumina paired-end (PE) sequencing data. Through bioinformatics analysis, the sequence was compared with other known cp genome sequences. The information helped us determine the phylogeny of this species.

## Materials and methods

### Sampling, DNA extraction, sequencing, and assembly

*A*. *venetum* seeds were collected from wild plants in Shaya County in the Xinjiang Uygur Autonomous Region, China (40°92´N, 82°21´E; 957 m). After removal of the bracts, seeds were surface sterilized for 1 min in 75% ethanol (v/v), rinsed three times with distilled water, and then germinated at 25°C in the dark on filter paper dampened with distilled water. When the plumule emerged, uniform seedlings were transplanted into plugged holes in plastic containers (5 cm × 5 cm × 5 cm, 1 seedling/container) filled with vermiculite and watered with modified Hoagland nutrient solution containing 2 mM KNO_3_, 0.5 mM NH_4_H_2_PO_4_, 0.25 mM MgSO_4_·7H_2_O, 0.1 mM Ca(NO_3_)_2_·4H_2_O, 50 μM Fe-citrate, 92 μM H_3_BO_3_, 18 μM MnCl_2_·4H_2_O, 1.6 μM ZnSO_4_·7H_2_O, 0.6 μM CuSO_4_·5H_2_O and 0.7 μM (NH_4_)_6_Mo_7_O_24_·4H_2_O. Solutions were renewed every 3 d. Seedlings were grown in a greenhouse at a temperature of 28°C/23°C (day/night) and photoperiod of 16:8 h (light:dark). The flux density was approximately 800 μmol m^−2^ s^−1^) and the relative humidity was 65%. Fresh leaves were collected on October 18, 2019, frozen in liquid nitrogen and then stored at −80°C until analysis [42].

Genomic DNA was isolated by the modified CTAB method. Agarose gel electrophoresis and a one drop spectrophotometer (OD-1000, Shanghai, China) were used to detect DNA integrity and quality. One library (250 bp) was constructed using pure DNA according to the manufacturer’s instructions (NEBNext^®^ UltraTM DNA Library Prep Kit for Illumina^®^). The library was constructed with an Illumina NovaSeq platform (Benagen Tech Solution Co. Ltd., Wuhan, China) and 150-bp paired-end reads were generated. The Illumina PCR adapter reads, low-quality reads and reads containing more than 5% unknown nucleotides “Ns” were filtered from the paired-end raw reads in the quality control step. All good-quality paired clean reads were obtained using SOAPnuke software (version: 1.3.0). The assembled reads were joined into a bidirectional iterative derivation using NOVOPlasty (version:3.13.1, parameter:k-mer = 127) to obtain the whole-genome sequence. The cp-like reads were used to assemble sequences using NOVOPlasty. NOVOPlasty assembled the partial reads and stretched as far as possible until a circular genome was formed. All circled sequences were searched by BLASTN (version: BLAST 2.2.30+, E-value ≤ 1^e-5^) against the reference database. Sequences with alignment greater than 1,000 bp and coverage greater than 90% were retained. Based on the depth of sequencing, PE reads alignment, and alignment with closely species to *A*. *venetum*, the candidate sequences were connected in order to determine whether they formed a loop. When a gap (including N sequence) appeared, Gapcloser (Version: 1.12) was used to fill in the hole to obtain the final splicing result [43]. After filtering the repeated sequences and the sequences with lengths less than 300 bp, 48 sequences with start codons of ATG, TTG, CTG, ATT, ATC, GTG, and ATA and end codons of TGA, TAG, and TAA, were retained to conduct subsequent analysis.

### Annotation and analysis of the cpDNA sequences

The cp genome sequence was annotated using the DOGMA program (http://dogma.ccbb.utexas.edu/) [44], and the tRNAscan-SE program was used to predict tRNAs in the genome [45]. The circular maps were drawn by the OGDRAWv1.2 program [46] (http://ogdraw.mpimp-golm.mpg.de/). In order to eliminate the influence of amino acid composition on codon usage, the characteristics of the variations in synonymous codon usage, the relative synonymous codon usage values (RSCU), base composition and codon content were analyzed using MEGA 7.0. Simple sequence repeats (SSRs) in the cp genome were identified using SSRHunter software (http://www.biosoft.net) [47, 48]. The parameters were set to five repeat units for mononucleotide SSRs, five repeat units for dinucleotide SSRs, three repeat units for trinucleotide SSRs, and three repeat units each for tetranucleotides, and pentanucleotide SSRs.

## Genome comparison

The pairwise alignments of cp genomes was conducted by MUMmer [42]. The mVISTA software was used to compere the *A*. *venetum* cp genome with three other cp genomes. *Nicotiana attenuata*, *Gossypium hirsutum*, and *Arabidopsis thaliana* (NC_035952.1, DQ345959, and NC_000932.1, respectively) using the annotation of *Sophora japonica* L. as reference [44, 45]. We determined the repeat structure, including forward and reverse repeats, using the REPuter software [46–49].

### Phylogenetic analysis

We downloaded 21 cp genome sequences from the NCBI organelle genome and nucleotide resource database, and used all genomes for phylogenetic analysis. Clustalw2 software (Conway Institute of Biomolecular and Biomedicine, Dublin, Ireland) was used to sequence the genome [50–53]. We used MEGA7.0 to analyze and draw a phylogenetic tree with ML (maximum likelihood). Bootstrap analysis was performed using 1,000 repetitions and TBR branch exchanges [54–56]. We used 1,000 replicates and TBR branch exchanges to complete the bootstrap analysis.

## Results

### Features of *A*. *venetum* cpDNA

**Fig 1:**
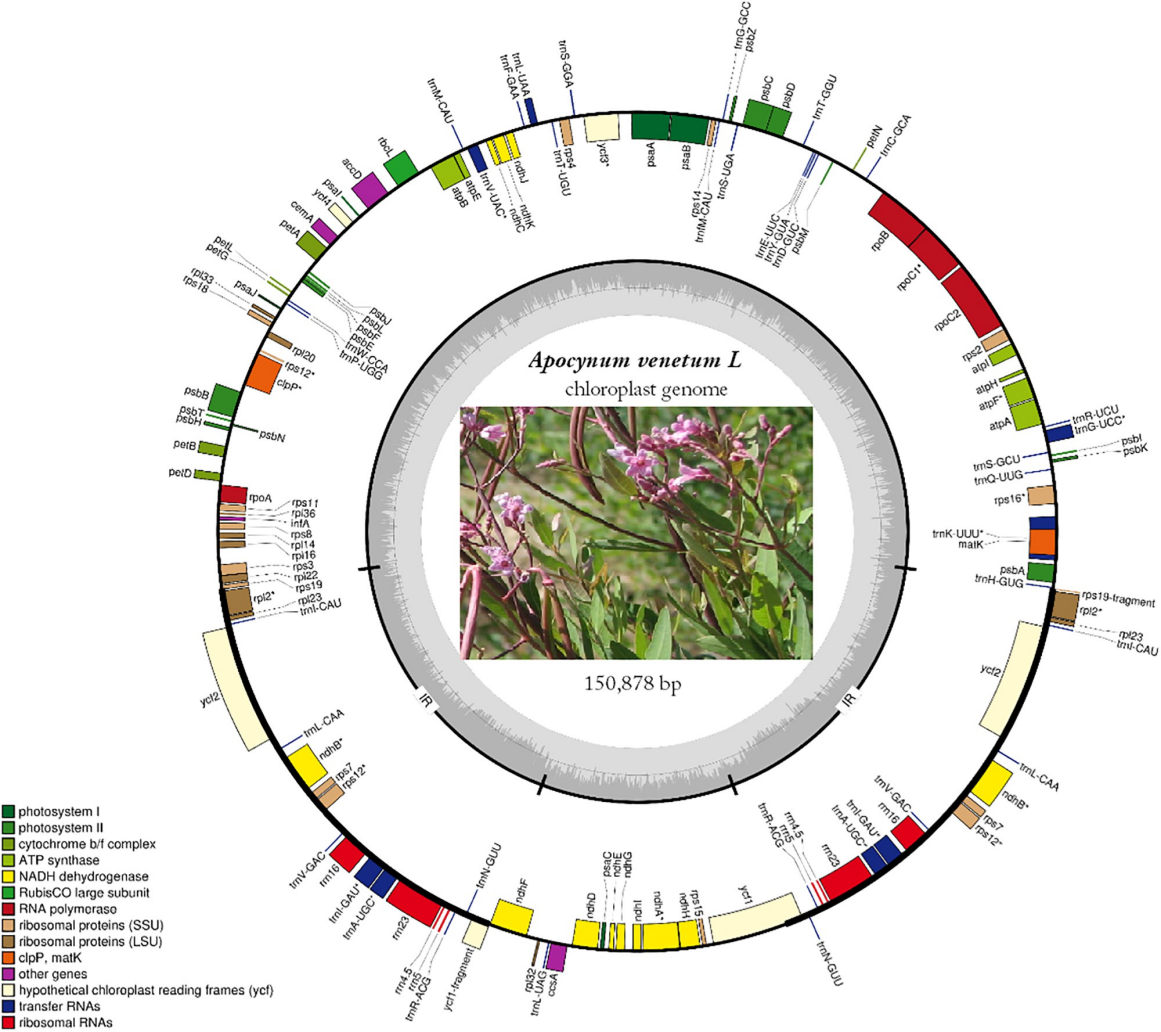
Map of *A*. *venetum* cpgenome. Thick lines indicate the extent of the inverted repeat regions (Ira and Irb), which separate the genome into small (SSC) and large (LSC) single copy regions. Genes drawn inside the circle are transcribed clockwise, and those outside are transcribed counterclockwise. Different colors represent different functional groups of genes.

The complete cp genome of *A*. *venetum* is 150,878 bp in length (GenBank accession number: MT568765) (Fig 1), and includes a pair of inverted repeats (IR) 25,810 bp long, separated by a large single region (LSC) and a small copy region (SSC) of 81,951 bp and 17,307 bp, respectively (Table 1). It is similar to the cp genome of other *Apocynaceae* species [57]

**Table 1:** Base composition in the *A*. *venetum* chloroplast genome.

Region	Length	A (%)	T (%)	C (%)	G (%)	AT (%)	GC (%)
Total_genome	150878	30.43	31.21	19.52	18.83	61.64	38.35
LSC	81951	31.02	32.49	18.69	17.8	63.51	36.49
IRA	25810	28.59	28.11	20.84	22.46	56.7	43.3
SSC	17307	33.85	33.65	17.09	15.32	67.49	32.41
IRB	25810	28.11	28.59	22.46	20.84	56.7	43.3

In the *A*. *venetum* cp genome, 131 functional genes were predicted, including eight rRNA genes, 37 tRNA genes, and 86 protein-coding genes (Table 2) Cp genomes in the IR regions include 33 duplicated genes, with approximately 15 tRNA genes (tRNAs), eight rRNA genes (rRNAs), and nine protein-coding genes (PCGs) (Fig 1). The LSC region includes 58 protein-coding and 22 tRNA genes, while the SSC region includes one tRNA gene and 11 protein-coding genes.

**Table 2:** Genes present in the *A*. *venetum* chloroplast genome.

Category for genes	Group of genes	Name of genes
Transcription and translation-related genes	transfer RNAs	trnM-CAU, trnR-ACG, trnY-GUA, trnG-UCC, trnL-UAG, trnI-GAU, trnW-CCA, trnR-UCU, trnQ-UUG, trnL-UAA, trnS-GGA, trnH-GUG, trnT-GGU, trnT-UGU, trnP-UGG, trnK-UUU, trnN-GUU, trnG-GCC, trnI-CAU, trnD-GUC, trnF-GAA, trnS-GCU, trnS-UGA, trnfM-CAU, trnE-UUC, trnV-GAC, trnA-UGC, trnV-UAC, trnL-CAA, trnC-GCA
	RNA polymerase	rpoB, rpoA, rpoC1, rpoC2
	ribosomal proteins(SSU)	rps8, rps4, rps16, rps14, rps7, rps12, rps2, rps11, rps19-fragment, rps19, rps18, rps3, rps15
	ribosomal proteins(LSU)	rpl2, rpl23, rpl32, rpl33, rpl36, rpl14, rpl16, rpl22, rpl20
	Translational initiation factor	infA
	ribosomal RNAs	rrn4.5, rrn5, rrn23, rrn16
Photosynthesis-related genes	NADH dehydrogenase	ndhA, ndhH, ndhF, ndhJ, ndhE, ndhI, ndhG, ndhK, ndhC, ndhD, ndhB
	photosystem I	psaI, psaJ, psaC, psaB, psaA
	photosystem II	psbA, psbL, psbF, psbB, psbK, psbJ, psbM, psbT, psbE, psbD, psbC, psbH, psbI, psbN, psbZ
	cytochrome b/f complex	petL, petN, petB, petG, petA, petD
	RubisCO	rbcL
	ATP synthase	atpA, atpE, atpH, atpI, atpB, atpF
	hypothetical chloroplast reading frames(ycf)	ycf2, ycf4, ycf1, ycf3, ycf1-fragment
Other genes	Maturase	matK
	Protease	clpP
	Envelope membrane protein	cemA
	Subunit of Acetyl-CoA carboxylase	accD
	C-type cytochrome synthesis gene	ccsA

The tRNA and protein-encoding gene sequences of the *A*. *venetum* cp were analyzed, and the codon usage frequency of the cp genome of *A*. *venetum* was inferred and summarized. A total of 17,318 codons represent the coding ability of 86 protein-coding genes and tRNA genes of *A*. *venetum* (Table 4), of which 1,814 codons code for leucine (10.47%), and 319 codons code for tryptophan (1.84%), which are the most common and least common amino acids in the cp genome of *A*. *venetum*, respectively. Codons ending in A and U are very common. Except for trtl-caa, all preferred synonymous codons (RSCU > 1) end in A or U. There are 14 intron-containing genes, including nine protein-coding genes and five tRNA genes (Table 3). Twelve genes (seven protein-coding and five tRNA genes) contain an intron, and two genes (ycf3 and clpP) contain two introns of the intragene region (Table 3). The size of the intron-containing matK gene in the trnK-UUU gene was 2,474 bp. The Rps12 gene is a trans-splicing gene with the 5’ end in the LSC region and the 3’ end in the IR region.

**Table 3:** Length of exons and introns in genes with introns in the *A*. *venetum* chloroplast genome.

Gene	Location	Exon I (bp)	Intron I (bp)	Exon II (bp)	Intron II (bp)	Exon III (bp)
trnK-UUU	LSC	35	2474	37		
rps16	LSC	226	837	41		
trnG-UCC	LSC	23	672	48		
atpF	LSC	411	706	144		
rpoC1	LSC	1599	748	451		
ycf3	SSC	155	794	226	717	126
trnV-UAC	LSC	37	588	36		
rps12	LSC	114	536	234		
clpP	LSC	228	642	291	763	69
rpl2	IR	434	649	391		
ndhB	IR	756	685	777		
trnI-GAU	IR	37	952	35		
trnA-UGC	IR	38	817	35		
ndhA	SSC	545	1039	553		

### Comparative analysis of genomic structure

**Fig 2:**
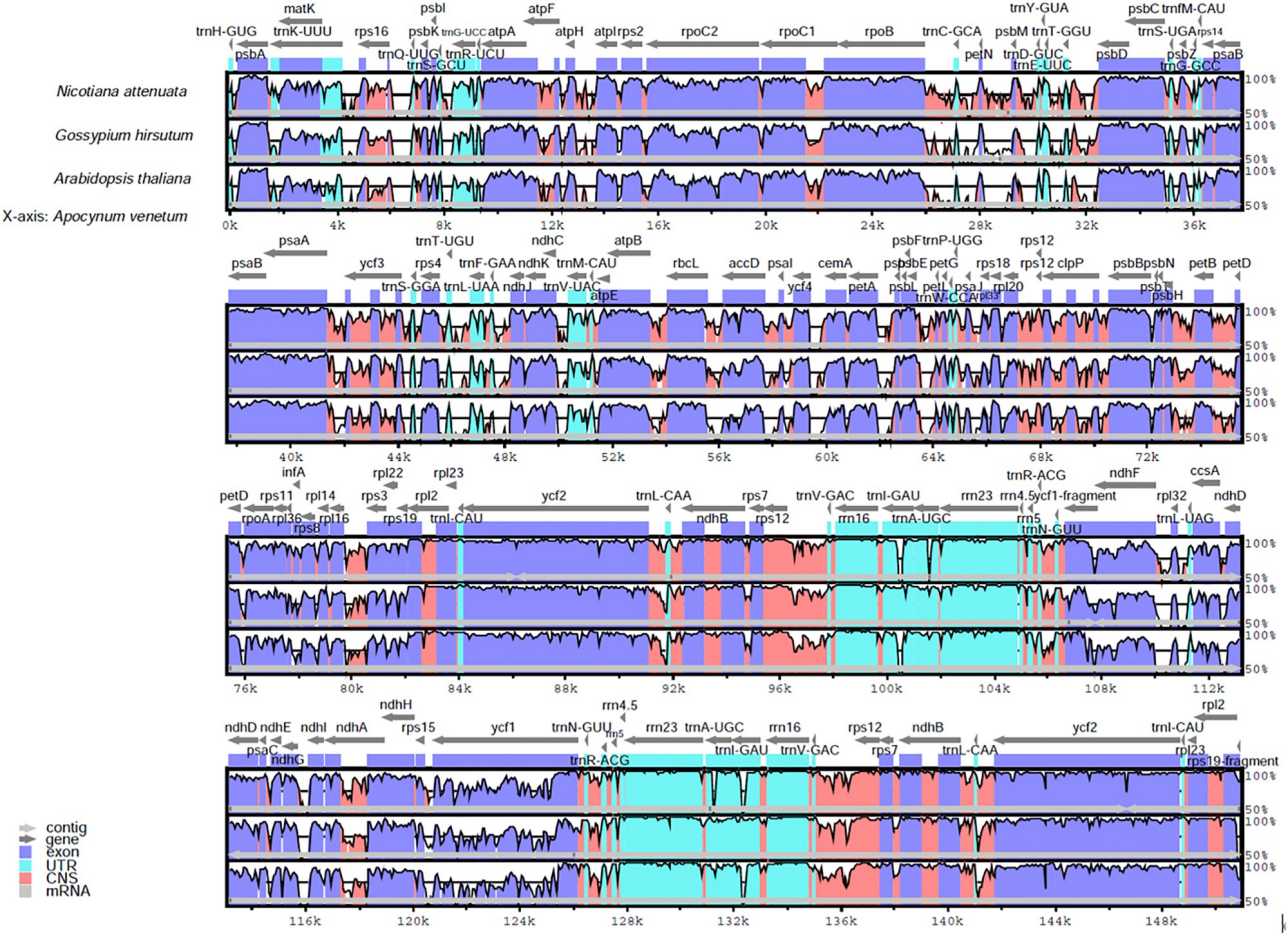
Comparison of the cpgenome sequences of four plants. Comparison of the cp genome sequences of *N attenuata*, *G*. *hirsutum*, *A*, *thaliana*, and *A*. *venetum* generated with mVISTA. Gray arrows indicate the position and direction of each gene. Red and blue areas indicate the intergenic and genic regions, respectively. The vertical scale indicates the percentage of identity, ranging from 50% to 100%.

Comparative genome analysis permits the examination of how DNA sequences diverge among related species. The whole cp genome sequence of *A*. *venetum* was compared to the sequences of *N*. *attenuata*, *G*. *hirsutum*, and *A*. *thaliana*. The identities of the entire sequence of the four cp genomes were drawn using the annotation mVISTA *N*. *attenuata* as a reference (Fig 2). The variation of the LSC and SSC regions were significantly greater than that of the IR regions. Moreover, the coding regions were more conserved than the non-coding regions. The most divergent coding regions of the four cp genomes were rnH-psbA, psbM-petN, trnC-GCA-petN, trnE-UUC-rpoB, trnY-GUA-trnE-UUC, trnV-UAC-ndhC, rbcL-accD, accD- psaI, LSC rpl32-trnL-UAG, and ndhI-ndhG ycf1-rps15 SSC, and the distribution of plastid rRNAs (rrn4.5, rrn5, rrn16, and rrn23) was the most conserved.

### Repeat sequence analysis

We studied the type, existence, and distribution of SSR in the cp genome of *A*. *venetum*. A total of 273 SSRs were found in *A*. *venetum*, most of which were distributed in LSC and SSC, and some in IR. These included 105 single nucleotide SSRs (38.46%), 142 dinucleotide SSRs (50.01%), 10 trinucleotides, 14 tetranucleotides, and two pentanucleotide repeats. The mononucleotide A and T repeat units accounted for the largest portion.

### Phylogenetic analysis

**Fig 3:**
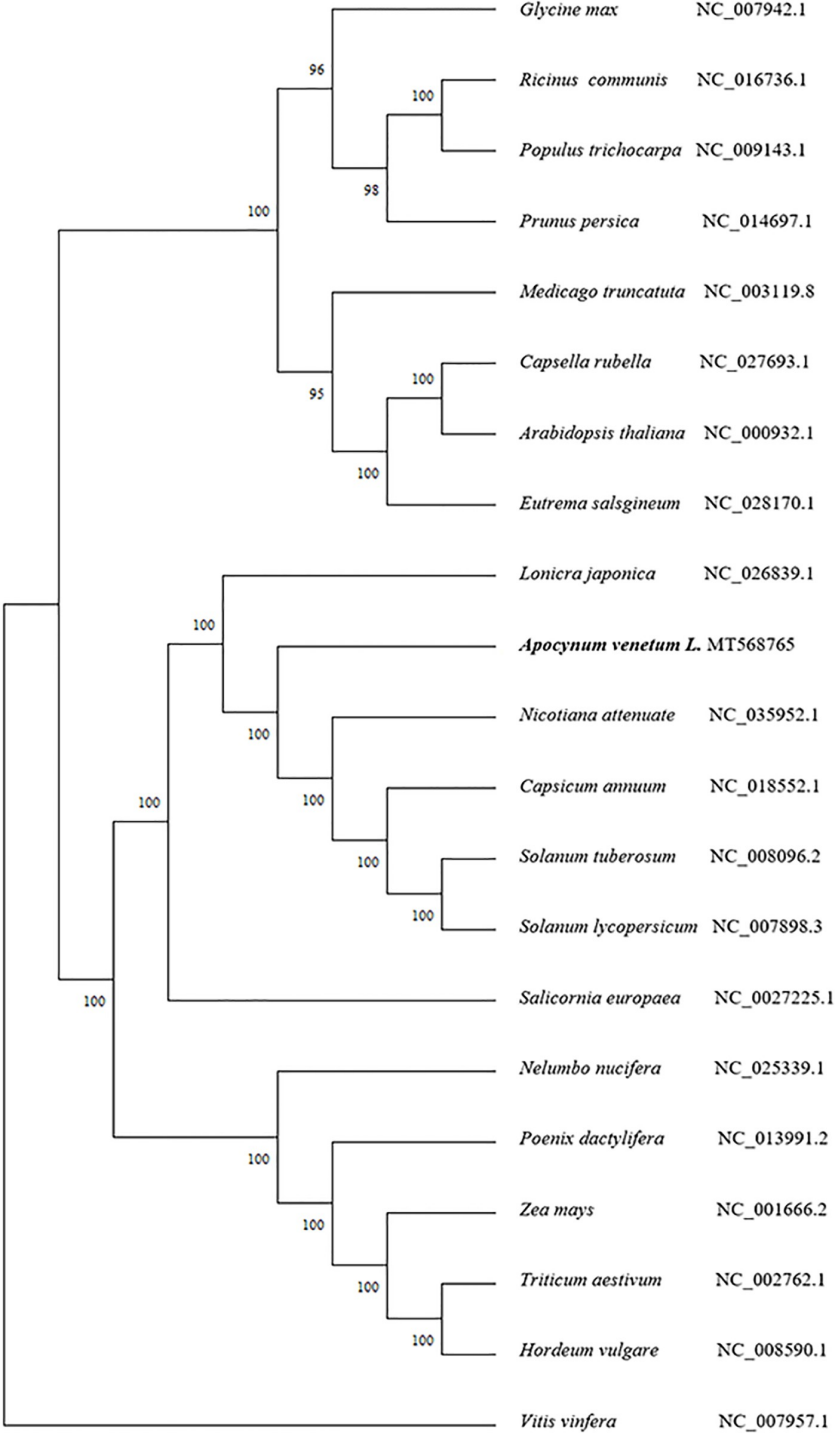
Phylogenetic tree analysis of whole chloroplast genome. Maximum likelihood (ML) phylogenetic tree reconstruction including 21 species based on all chloroplast genomes. The bootstrap value, based on 1,000 replicates, is shown on each node. *V*. *vinfera* was used as the outgroup. The GenBank accession numbers are listed following the species name.

The cpDNA gene content is highly conserved in most land plants. We downloaded 21 complete cp genome sequences from the NCBI Organelle Genome Resources database to reveal the phylogenetic location of *A*. *venetum* (Fig 3). In this study, we constructed a phylogenetic tree to infer the phylogenetic positions of *A*. *venetum* cp genomes. The evolutionary tree was separated into four clusters. The phylogenetic tree showed that *Vitis vinfera* were clustered on a single terminal branch. Phylogeny analysis showed that *Glycine max*, *Ricinus communis*, *Populus trichocarpa*, *Prunus persica*, *Medicago truncatuta*, *Capsella rubella*, *A*. *thaliana*, and *Eutrema salsgineum* formed an independent branch. We found that *A*. *venetum* L. was grouped into a terminal branch with *Lonicra japonica* and *N*. *atienuate*, *Capsicum annuum*, *Solanum tuberosum*, *Solanum lycopersicum* and *Salicornia europaea*. *Meanwhile*, *Nelumbo nucifera*, *Poenix dactylifera*, *Zea mays*, *Triticum aestivum*, and *Hordeum vulgare* were clustered on a branch.

## Discussion

In this study, we assembled, annotated and analyzed the complete cp sequence of *A*. *venetum*. We then analyzed its features, GC content, gene structure, and repeat sequences. The complete cp genome of *A*. *venetum* has a total length of 150,878 bp, with a pair of IRs of 25,810 bp that separate an LSC region of 81,951 bp and an SSC region of 17,307 bp. The DNA GC content of LSC, SSC, IR, and the whole genome were 36.49%, 32.41%, 43.3%, and 38.36%, respectively, which were similar to those of other species in *Nerium*. DNA GC content is an important index to evaluate the genetic relationship of *Nerium oleracea*, and the cpDNA GC content of *Nerium indicum* is similar to that in other species of *Apocynaceae* [58–63]. The content of DNA GC in the IR region is higher than that in other regions (LSC, SSC); this phenomenon is common in other plants [64–66]. The relatively high DNA GC content in the IR region was mainly attributed to the rRNA gene and the tRNA gene [67, 68].

Cp sequences have been used to compare the genetics of plant species, gene flow between species, and the size of ancestral populations of sister species [69]. Therefore, it is necessary to understand cp differences among species. We observed the order of approximately the same genes and the coding regions in the organization of the cp genome (Fig 2). The cp genome is considered to be highly conservative compared to the non-coding region, and the two infrared regions are less divergent than the LSC and SSC regions. The four cp genomes with the most different coding regions (rnH-psbA, psbM-petN, trnC-GCA-petN, trnE-UUC-rpoB, trnY-GUA-trnE-UUC, trnV-UAC-ndhC, rbcL-accD, accD-psaI, LSC rpl32-trnL-UAG, and ndhI-ndhG ycf1-rps15 SSC) and the four ribosomal RNA genes (rrn4.5, rrn5, rrn16, and rrn23) were the most conserved. Similar results have been observed in other plant cp genomes.

Cp genomes are highly conserved and contain a large amount of genetic information. The noncoding regions are less conserved than the coding regions [70, 71]. The genes trnK-UUU, rps16, trnG-UCC, atpF, rpoC1, trnV-UAC, rps12, rpl2, ndhB, trnI-GAU, trnA-UGC, and ndhA have one intron each, while clpP and ycf3 contain two introns. A trans-splicing event was also observed in the rps12 gene (Table 4). Previous studies have reported that ycf3 is necessary for the stable accumulation of photosystem I complexes [42, 72]. Therefore, we believe that the intron gain in ycf3 of *A*. *venetum* provides insight into the evolution of photosynthesis. As cp-specific SSRs are inherited from one parent and are mainly formed by the chain mismatch caused by the sliding of polymerase during DNA replication, they are often used in population genetics, species identification, and evolutionary process research on wild plants. In addition, the cp genome sequence is highly conserved, and SSR primers of cp genome can be transferred across species and genera. There were 273 SSRs detected in in the CP genome of *A*. *venetum*. Among these SSRs, mono-, di-, tri-, tetra-, and pentanucleotide were detected. The average density of SSRs was 1.809 SSR/kb in *A*. *venetum* (A/T as the main component). These cpSSR markers could be used for future studies of the genetic structure, diversity, and differentiation of *A*. *venetum* and its related species.

**Table 4:** Codon-anticodon recognition patterns and codon usage of the *A*. *venetum* chloroplast genome.

Amino Acid	Codon	Number	RSCU*	tRNA	Amino Acid	Codon	Number	RSCU*	tRNA
Stop	UAA	23	1.53		Met	AUG	401	1	trnM-CAU
Stop	UAG	9	0.6		Asn	AAU	627	1.56	
Stop	UGA	13	0.87		Asn	AAC	178	0.44	
Ala	GCU	459	1.78		Pro	CCU	270	1.49	
Ala	GCC	174	0.67		Pro	CCC	153	0.84	
Ala	GCA	281	1.09		Pro	CCA	191	1.05	trnP-UGG
Ala	GCG	118	0.46		Pro	CCG	111	0.61	
Cys	UGU	128	1.44		Gln	CAA	495	1.54	trnQ-UUG
Cys	UGC	50	0.56	trnC-GCA	Gln	CAG	148	0.46	
Asp	GAU	578	1.59		Arg	CGU	227	1.32	trnR-ACG
Asp	GAC	147	0.41	trnD-GUC	Arg	CGC	78	0.45	
Glu	GAA	682	1.51	trnE-UUC	Arg	CGA	240	1.39	
Glu	GAG	220	0.49		Arg	CGG	90	0.52	
Phe	UUU	611	1.28		Arg	AGA	285	1.65	trnR-UCU
Phe	UUC	343	0.72	trnF-GAA	Arg	AGG	115	0.67	
Gly	GGU	412	1.28		Ser	UCU	366	1.67	
Gly	GGC	144	0.45	trnG-GCC	Ser	UCC	223	1.02	trnS-GGA
Gly	GGA	474	1.48		Ser	UCA	246	1.12	trnS-UGA
Gly	GGG	253	0.79		Ser	UCG	131	0.6	
His	CAU	315	1.45		Ser	AGU	269	1.23	
His	CAC	120	0.55	trnH-GUG	Ser	AGC	81	0.37	trnS-GCU
Ile	AUU	722	1.5		Thr	ACU	353	1.63	
Ile	AUC	294	0.61	trnI-CAU	Thr	ACC	176	0.81	trnT-GGU
Ile	AUA	431	0.89		Thr	ACA	238	1.1	trnT-UGU
Lys	AAA	594	1.49		Thr	ACG	98	0.45	
Lys	AAG	204	0.51		Val	GUU	361	1.49	
Leu	UUA	597	1.97		Val	GUC	114	0.47	trnV-GAC
Leu	UUG	365	1.21	trnL-CAA	Val	GUA	360	1.48	
Leu	CUU	388	1.28		Val	GUG	135	0.56	
Leu	CUC	107	0.35		Trp	UGG	319	1	trnW-CCA
Leu	CUA	225	0.74		Tyr	UAU	512	1.64	
Leu	CUG	132	0.44		Tyr	UAC	114	0.36	trnY-GUA

RSCU *: relative synonymous codon usage.

The phylogenetic positions of 21 cp genomes were successfully analyzed with the support of full bootstrap at almost all nodes. A phylogenetic tree was constructed for the data by ML, and *V*. *vinfera* was used as an outgroup. In this method, an initial tree is first built using a fast but suboptimal method such as the neighbor-joining method, and its branch lengths are adjusted to maximize the likelihood of the data set for that tree topology under the desired model of evolution. The results show that *A*. *venetum* has the closest relationship with *L*. *japonica*, *N*. *attenuata*, *C*. *annuum*, *S*. *tuberosum*, and *S*. *lycopersicum*.

## Conclusion

We analyzed and illustrated the complete cp genome of *A*. *venetum*. The cp genome is conservative and similar to other species of *Apocynum*. These results provide a reference for the complete assembly of the cp genome of *Apocynaceae*, which may aid future breeding and research efforts. It may also assist in the development of unique *Apocynaceae* DNA barcodes of *Apocynaceae* and in determining the evolutionary history of *Apocynaceae*.

## Supporting information

S1 File (ZIP)

S2 File (RAR)

S3 File (ZIP)
