# Supplementary material for: The chloroplast genome sequence and phylogenetic analysis of Apocynum venetum L
Source: PLoS One. 2022 Mar 10;17(3):e0261710. doi: 10.1371/journal.pone.0261710 (PMC8912179; doi:10.1371/journal.pone.0261710)
Supplement: Attachment — Submitted filename: rebuttal letter2021.10.9.docx [file pone.0261710.s004.docx]

Dear Editors and Reviewers:

Thank you for your advice and for the reviewers’ comments concerning our manuscript entitled “The chloroplast genome sequence and phylogenetic analysis of *Apocynum venetum* L”. Those comments are all valuable and very helpful for revising and improving our paper, in addition to their important guiding significance for our research. We have studied the comments carefully and have made corrections, which we hope will meet with approval. The revised portions are marked in red in the paper. The main corrections in the paper and the responses to the editor’s and reviewers’ comments are as follows:

Responses to the editor’s comments:

1. Please ensure that your manuscript meets PLOS ONE's style requirements, including those for file naming. The PLOS ONE style templates can be found at <https://journals.plos.org/plosone/s/file?id=wjVg/PLOSOne_formatting_sample_main_body.pdf> and <https://journals.plos.org/plosone/s/file?id=ba62/PLOSOne_formatting_sample_title_authors_affiliations.pdf>.

Response: Thank you very much for your comments, which helped to improve our manuscript. We have revised the manuscript accordingly.

2. We note that the grant information you provided in the ‘Funding Information’ and ‘Financial Disclosure’ sections do not match.

When you resubmit, please ensure that you provide the correct grant numbers for the awards you received for your study in the ‘Funding Information’ section.

Response: Thank you very much for your comments. We have revised the mistakes.

3. Thank you for stating the following in the Funding Section of your manuscript:

“The National Natural Science Foundation of China (Grant No. 31760242), Gansu Provincial Natural Science Foundation (Grant No. 20JR10RA120), Gansu Provincial Natural Science Foundation the Ministry of Education of China for an Innovative Research Team in University (IRT 17R88), and the Fundamental Research Funds for the Central Universities (Grant No. 31920190021)”.

We note that you have provided funding information that is not currently declared in your Funding Statement. However, funding information should not appear in the Funding section or other areas of your manuscript. We will only publish funding information present in the Funding Statement section of the online submission form.

Please remove any funding-related text from the manuscript and let us know how you would like to update your Funding Statement. Currently, your Funding Statement reads as follows:

“The funders had no role in study design, data collection and analysis, decision to publish, or preparation of the manuscript”.

Response: We are thankful for this suggestion. We apologize for our mistake, and we have deleted the funding information in the manuscript.

4. We note that you have stated that you will provide repository information for your data at acceptance. Should your manuscript be accepted for publication, we will hold it until you provide the relevant accession numbers or DOIs necessary to access your data. If you wish to make changes to your Data Availability statement, please describe these changes in your cover letter and we will update your Data Availability statement to reflect the information you provide.

Response: We are thankful for your suggestion. No changes were required.

5. PLOS requires an ORCID iD for the corresponding author in Editorial Manager on papers submitted after December 6th, 2016. Please ensure that you have an ORCID iD and that it is validated in Editorial Manager. To do this, go to ‘Update my Information’ (in the upper left-hand corner of the main menu), and click on the Fetch/Validate link next to the ORCID field. This will take you to the ORCID site and allow you to create a new iD or authenticate a pre-existing iD in Editorial Manager. Please see the following video for instructions on linking an ORCID iD to your Editorial.

Response: Thank you for your positive response to our work and the kind advice. We have linked an ORCID iD to the Editorial Manager.

6. Please amend the manuscript submission data (via Edit Submission) to include author Xiaonong Guo.

Response: Thank you very much for these comments. We have amended the manuscript submission data.

7. Thank you for submitting the above manuscript to PLOS ONE. During our internal evaluation of the manuscript, we found significant text overlap between your submission and the following previously published works, some of which you are an author.

https://www.researchgate.net/publication/322729499_Complete_Chloroplast_Genome_Sequence_and_Phylogenetic_Analysis_of_Paeonia_ostii

https://www.mdpi.com/1422-0067/19/8/2443/htm

We would like to make you aware that copying extracts from previous publications, especially outside the methods section, word-for-word is unacceptable. In addition, the reproduction of text from published reports has implications for the copyright that may apply to the publications.

Please revise the manuscript to rephrase the duplicated text, cite your sources, and provide details as to how the current manuscript advances on previous work. Please note that further consideration is dependent on the submission of a manuscript that addresses these concerns about the overlap in text with published work.

We will carefully review your manuscript upon resubmission, so please ensure that your revision is thorough.

Response: We apologize for our negligence and mistakes. We have checked throughout the text and corrected similar format issues. On the basis of the methods, we have reedited the contents and deleted the repetitive statements. Please see lines 111–134.

Responds to the reviewer’s comments:

Reviewer #1:

1. While the study appears to be sound, the language is unclear, making it difficult to follow. I advise the authors work with a writing coach or copyeditor to improve the flow and readability of the text.

Response: We have improved both the language and readability and sent our manuscript to LetPub for additional improvement of language and grammar.

2. The authors should carefully read and follow the PLOS ONE guidelines. There is no line number in the manuscript. Figures should be cited “Fig 1”, “Fig 2”, etc. References with more than six authors should list the first six author names, followed by “et al.”

Response: We apologize for our negligence and mistakes. We have checked throughout the text and corrected similar format issues. We added the line number in the revised manuscript. Thank you for pointing out this mistake in the figures. It has now been corrected. The reference format has also been corrected. Please see lines 387–388, 395–396, 519–522.

3. The authors should give more details for chloroplast genome assembly. How they defined the chloroplast-like reads? Please check the version of NOVOPlasty.

Response: According to your suggestion, we have described this method with more detail in the revised text. Please see lines 103–107. We have checked and corrected the version of NOVOPlasty. Please see line 99.

4. In Results, there are no results description in “Comparative Analysis of Genomic Structure” and “Phylogenetic Analysis”.

Response: Thank you for pointing this out. The mistake has now been corrected and this information has been added. Please see lines 185–196 and 206–219.

5. In Discussion, authors should avoid the first paragraph, they are part of the Introduction. And, authors need to emphasize the novel insights obtained from their study.

Response: Thank you for your advice. We modified the text according to your suggestion.

6. The authors said “used 13 chloroplast genome sequences for phylogenetic analysis” in Page 5, but there are 21 complete chloroplast genome sequences in Page 10 and Figure 3. Please check and correct it.

Response: We apologize for our negligence and mistakes. You are absolutely right about the number of chloroplast genome sequences for phylogenetic analysis, and we thank you for pointing out this mistake, which we have corrected. Please see line 136.

7. Which software was used to draw the phylogenetic tree? The authors said “used MEGA7.0 to analyze and draw a phylogenetic tree with ML (maximum likelihood)” in method. However, in Discussion was “the Bayesian Inference (BI) method based on RAxML was used to construct a phylogenetic tree”. Please carefully check.

Response: Thank you for pointing this out. We only used MEGA7.0 to analyze and draw a phylogenetic tree with ML (maximum likelihood). We have fixed the mistake at lines 274–278.

8. The reviewer suggested the authors to remove the Table 2.

Response: Thank you for your advice. We have removed it from our revised manuscript. Please see line 161.

9. In page 12, the authors mentioned the reference 74 in “Previous studies have reported that ycf3 is necessary for the stable accumulation of photosystem I complexes [42, 74]”, but missed it in References.

Response: Thank you for pointing out this mistake about the reference. According to the reviewer’s suggestion, we have added the mentioned literature in the reference section. Please see lines 544–546.

Reviewer #2: This manuscript “The chloroplast genome sequence of *Apocynum venetum* L.” reported the chloroplast genome sequence of *Apocynum venetum*, and provided some basic information about this genome, such as GC contents, LSC, SSC and coding gene information, which will be helpful for the studies in plant evolution. However, the written is very poor, and it is very descriptive that missing some very important information. For example, the author claim that the *A. venetum* chloroplast genome encodes 131 genes, including 86 protein-coding genes, 8 ribosomal RNA genes, and 37 transfer RNA genes. Is there any novel genes? How about this gene expression pattern, especially when plants meet the stress? The author could also compare the protein amount of chloroplast genes in some relative plant species and try to explain why *Apocynum venetum* could grow in such dry condition.

Response: Thank you very much for this insightful comment. The article has been polished by a professional editing company. At present, we could not detect novel genes in the chloroplast genome sequence of *Apocynum venetum.* In the future, we will excavate the stress-resistant genes and study their stress-resistance mechanisms in combination with transcriptome and physiological experiments.
