# Supplementary material for: The chloroplast genome sequence and phylogenetic analysis of Apocynum venetum L
Source: PLoS One. 2022 Mar 10;17(3):e0261710. doi: 10.1371/journal.pone.0261710 (PMC8912179; doi:10.1371/journal.pone.0261710)
Supplement: Attachment — Submitted filename: Response to Reviewers.docx [file pone.0261710.s005.docx]

Dear Editors and Reviewers:

Thank you for your letter and comments on our manuscript. These comments helped us improve our manuscript, and provided important guidance for future research.

We have addressed the editor’s and reviewers’ comments to the best of our abilities, and revised text to meet PLOS ONE style requirements. We hope this meets requirements for a publication.

The revised portions are marked in red in the manuscript. The main comments and our specific responses are detailed below:

Responses to the editor’s comments:

we feel that it has merit but does not fully meet PLOS ONE’s publication criteria as it currently stands. Therefore, we invite you to submit a revised version of the manuscript that addresses the points raised during the review process.

Response: Thank you very much for your comments, we carefully checked the format of references and improved references 2-4, 24 and 27, which are in lines 272-277 and 348-358 respectively; It was further verified that references 13 and 22 were adjusted interchangeably according to the citation, in lines 305-310 and 338-343, respectively, and reference 42 replaced a more appropriate reference. The details are in lines 400-405.

Please include the following items when submitting your revised manuscript:

- A rebuttal letter that responds to each point raised by the academic editor and reviewer(s). You should upload this letter as a separate file labeled 'Response to Reviewers'.
- A marked-up copy of your manuscript that highlights changes made to the original version. You should upload this as a separate file labeled 'Revised Manuscript with Track Changes'.
- An unmarked version of your revised paper without tracked changes. You should upload this as a separate file labeled 'Manuscript'.

Response: The above three documents are named as required

Reviewer #1:

The new version of the manuscript has revised most of the comments and concerns that I rose in my previous review. However, there are many English language errors throughout the article. The author doesn't write seriously，there are many errors need to be corrected.

Response: Thanks for the constructive suggestions to improve our manuscript. We commissioned letpub, a professional editing agency, to edit the full text of the article, and attach the letpub editing certificate.

Many references listed in the manuscript are not quoted accurately. For example, the references 50-53 and 54-56 in lines 129 and 131 respectively, these citations does not appear to have any context to the text. Moreover, the references are not numbered in the order in which they appear in the text.

Response: We are very sorry that some references were cited inappropriately due to our negligence. For 50-56 references, we selected the appropriate ones and replaced them one by one. See lines 427-462for details. secondly, we carefully checked the format of references and improved references 2-4, 24 and 27, which are in lines 272-277 and 348-358 respectively; It was further verified that references 13 and 22 were adjusted interchangeably according to the citation, in lines 305-310 and 338-343, respectively, and reference 42 replaced a more appropriate reference. The details are in lines 400-405.

The cp genome sequence of *A. venetum* (MT568765) was not found in GenBank.

Response: Since our article has not been published yet and the data has not been released yet, it cannot be searched on GenBank. This article is currently under minor revision. The data we communicate with ncbi will be released on December 6th.

Reviewer #2:

The authors have addressed all questions that I concerned. I recommend to accept this manuscript in this version.

Response: Thank you very much for your comments.
